# Generating Political Priority for the Health Needs of the 21st Century: A Qualitative Policy Analysis on the Prioritization of Rehabilitation Services in Uganda

**DOI:** 10.34172/ijhpm.8347

**Published:** 2024-07-22

**Authors:** Rachel Neill, Elizeus Rutebemberwa, Raymond Tweheyo, Sam Tukei Ojulo, Gerald Okello, Abdulgafoor M. Bachani, Yusra Ribhi Shawar

**Affiliations:** ^1^Johns Hopkins International Injury Research Unit, Health Systems Program, Department of International Health, Johns Hopkins Bloomberg School of Public Health, Baltimore, MD, USA; ^2^College of Health Sciences, School of Public Health, Makerere University, Kampala, Uganda; ^3^Department of International Health, Johns Hopkins University Blomberg School of Public Health, Baltimore, MD, USA; ^4^Paul H. Nitze School of Advanced International Studies, Johns Hopkins University, Washington, DC, USA

**Keywords:** Health Policy, Health Systems, Disability, Rehabilitation, Africa, Uganda

## Abstract

**Background:**

Few low- or middle-income countries (LMICs) have prioritized the expansion of rehabilitation services. Existing scholarship has identified that problem definition, governance, and structural factors are influential in the prioritization of rehabilitation. The objective of this study was to identify the factors influencing the prioritization and implementation of rehabilitation services in Uganda.

**Methods:**

A case study design was utilized. The Prioritization of Rehabilitation in National Health Systems framework guided the study. Data sources included 33 key informant interviews (KIIs) with governmental and non-governmental stakeholders and peer-reviewed and grey literature on rehabilitation in Uganda. A thematic content analysis and concept map were conducted to analyze the data.

**Results:**

Rehabilitation is an unfunded priority in Uganda, garnering political attention but failing to receive adequate financial or human resource allocation. The national legacy of rehabilitation as a social program, instead of a health program, has influenced its present-day prioritization trajectory. These include a fragmented governance system, a weak advocacy coalition without a unified objective or champion, and a lack of integration into existing health systems structures that makes it challenging to scale-up service provision. Our findings highlight the interactive influences of structural, governance, and framing factors on prioritization and the importance of historical context in understanding both prioritization and implementation.

**Conclusion:**

Our findings demonstrate challenges in prioritizing emerging, multi-sectoral health areas like rehabilitation. Strategic considerations for elevating rehabilitation on Uganda’s policy agenda include generating credible indicators to quantify the nature and extent of the population’s need and uniting governmental and non-governmental actors around a common vision for rehabilitation’s expansion. We present opportunities for strengthening rehabilitation, both in Uganda and in similar contexts grappling with many health sector priorities and limited resources.

## Background

**Key Messages**

**Implications for policy makers**

- Rehabilitation is an unfunded priority in Uganda, with prioritization and implementation limited by fragmented governance, conflicting problem definitions, and lack of integration into the health system.
- Aligning on a national rehabilitation strategic plan could assist policy-makers in bringing together a fragmented set of stakeholders into a unified policy coalition.
- Investing in the generation of credible indicators — including integrating rehabilitation into routine health management information systems and conducting population-based surveys to estimate unmet needs — is important to justify the allocation of scarce public funds.

**Implications for the public**

The political prioritization of health issues influences the structure of the healthcare system, the services that are available to the population, how much they cost, and their quality. Analyzing the political prioritization and implementation of rehabilitation can support the public in advocating for services that meet population needs. Among other findings, our research points to the importance influence of domestic coalitions — including disabled people’s organizations, civil society, families, and communities — in advancing the prioritization of rehabilitation. Our findings can be used as an advocacy tool to expand access to rehabilitation services for the Ugandan people and others in similar contexts.

Chronic diseases and injuries are now major contributors to global disability adjusted life years,^1^ leading to an increased need for rehabilitation services.^2^ Rehabilitation is defined by the World Health Organization (WHO) as, “a set of interventions designed to optimize functioning and reduce disability in individuals with health conditions in interaction with their environment.”^3^ The need for rehabilitation has garnered global attention via Rehabilitation 2030: A Call to Action,^4^ and the World Health Assembly’s Resolution 76.6 for strengthening rehabilitation in health systems.^5^ The World Rehabilitation Alliance has also emerged as a global advocacy body to champion rehabilitation.^6^ However, global initiatives have not translated into widespread prioritization of rehabilitation services in low- and middle-income countries (LMICs).^7^ Limited prioritization of rehabilitation is particularly acute in primary healthcare, both a critical entry point for the diagnosis and referral of conditions requiring rehabilitation and a potential way to bring rehabilitation services closer to communities.^8^

 Political prioritization is driven by the interplay of epidemiological and clinical evidence as well as political, bureaucratic, and social factors and power dynamics.^9^ Rehabilitation’s lack of prioritization mirrors many multisectoral health issues including non-communicable diseases,^10,11^ road traffic injuries,^12^ mental health,^13^ and urban health.^14^ Across these issues, political prioritization is challenged by a lack of credible indicators, fragmented governing coalitions, and unconvincing frames.^10-14^ However, there are also positive examples of multisectoral issues receiving high levels of political prioritization, such as the response to the HIV/AIDS epidemic,^15,16^ policies to support violence against women,^17,18^ and nutrition advocacy.^19^ These examples point to the importance of evidence, issue framing, and the role of domestic advocacy coalitions in priority setting.^15-19^

###  Study Setting: Rehabilitation in Uganda 

 Uganda is a relevant case to study the prioritization of rehabilitation. An estimated 12.4% of Ugandans are living with a disability,^20^ and an estimated 6.8M Ugandans could benefit from rehabilitation.^21^ Uganda has a long history of policy inclusion for persons with disabilities (PWD), setting it apart from many sub-Saharan African countries.^22^ This was influenced by civil society activism, the 1995 constitution and subsequent legal and policy frameworks, and the signing of international agreements on the rights of PWD.^20,22^

 Within this context, the prioritization of rehabilitation has ebbed and flowed, influenced by the development of community-based rehabilitation (CBR),^20,23^ civil conflict,^24-26^ and the priorities of domestic and transnational actors. Domestically, the primary responsibilities for policy-making and strategic direction of rehabilitation are vested in the Ministry of Health (MoH) and the Ministry of Gender, Labour, and Social Development (MoGLSD). The MoH manages national and regional referral hospitals while lower levels of the health system are overseen by district local governments.^27^ The CBR system is overseen by the MoGLSD and has largely focused on social rehabilitation. In addition, the Ministry of Education and Sports (MoE&S) is responsible for affirmative action programs for PWD, participation of PWD in education, and the management of specialized schooling for PWD.

 Transnational actors also have substantive influence on the health sector. In the 2022/2023 fiscal year, 42.1% of Uganda’s total current health expenditure came from external sources, compared to 17.1% from government, and 38.7% from households.^28^

###  Study Objectives 

 The objective of this study was to identify the factors influencing the prioritization and implementation of rehabilitation services in Uganda. To do this, we conducted a qualitative case study analysis utilizing empirical evidence from a document review and key informant interviews (KIIs).

## Methods

 We used a case study design, which is considered appropriate when investigating a single, in-depth phenomenon.^29^ The primary data source was KIIs. The secondary data source was a literature review, used to supplement the KII data, triangulate our findings, and add additional examples. We adhered to the Standards for Reporting Qualitative Research.^30^

###  Conceptual Framework 

 Neill et al^7^ developed an empirical framework that identified key factors driving the relative prioritization of rehabilitation in LMIC national health systems (Table 1), which we adopted to guide this study.

**Table 1:** Overview of the Framework for the Prioritization of Rehabilitation^7^

**Framework Component**	**Sub-component**	**Definition**
Problem definition	Problem clarity	Common understanding of the problem
	Solution acceptability	Reaching consensus on a set of acceptable and feasible solutions
Governance	Domestic advocacy coalitions	Cohesiveness, representativeness, and power of domestic stakeholders advancing rehabilitation
	Transnational actors	The role of non-domestic actors influencing the domestic context
Structural factors	National legacies	Political and historical contexts which influence decision-making and the rehabilitation system
	Health systems structures	Arrangements of health services, financing, and data systems

 Per the conceptual framework, we defined the prioritization of rehabilitation as, “concern for the issue, the enactment of policies that advance consensus-based solutions, and the consistent application of public funds aligning with the unmet need.”^7^ The categories of the framework guided the development of the KII guides, organized our analysis of literature data, and formed the initial set of deductive codes for thematic analysis.

####  Researcher Profiles and Reflexivity

 This research was conducted by a team of junior, mid-career, and senior Ugandan and non-Ugandan researchers working within the Learning, Acting, and Building for Rehabilitation in Health Systems consortium. Members of the research team had different academic training and professional backgrounds, including political science, public health, qualitative methods, and rehabilitation. Two individuals support rehabilitation services in Uganda, two individuals are public health researchers in Uganda, and three individuals are public health researchers in the United States.

 The research team worked collaboratively, engaging in bi-weekly meetings to design the study, conduct the document review, develop the KII guides, conduct interviews, and analyze the data. These regular discussions promoted reflexivity and allowed us to investigate different positionalities as Ugandans and non-Ugandans.

####  Literature Review 

 We collected documents using a purposeful search of peer reviewed and grey literature and policy documents related to rehabilitation and assistive technology in Uganda. We searched PubMed and Google Scholar using “rehabilitation,” “assistive technology,” “Uganda,” “policies,” “programs,” “health systems,” and “rehabilitation” as key words. We used the Google search engine to search for grey literature. Ugandan government agency websites were also consulted to locate policy documents. We did not set a date eligibility as we intended to use documents both to gain insight into current day prioritization and to construct a timeline of rehabilitation in Uganda.

 Document collection and analysis was iterative. We searched for and identified documents between April and December 2021. For each identified document, we read them in full, extracted details in notes, and consulted the reference lists to snowball additional documents. We stopped our literature search when we reached theoretical saturation – when sources contained few new relevant insights.^31^

####  Key Informant Interviews 

 We conducted 29 KIIs with 33 key informants (KIs) working in rehabilitation and deeply familiar with rehabilitation’s governance and implementation in Uganda (Table 2). An initial list of KIs was compiled via our knowledge of rehabilitation in Uganda and individuals and organizations identified in the literature review. We added two additional KIs based on feedback from participants. The KIs were disaggregated by stakeholder profile, and we included at least two individuals from each profile in our sample. Per the Sex and Gender in Research principles,^32^ we included male (n = 26) and female (n = 7) KIs.

**Table 2:** Key Informant Interview Profiles and Number

**Profile**	**Number of Participants**
National Government, including representatives from executive agencies and the legislative branch	6
District Government Health Officers	5
Public hospital managers and clinicians	4
Private not-for-profit hospitals managers and clinicians	2
Rehabilitation health professionals’ association	3
NGOs	7^a^
Organizations representing PWD	2
Rehabilitation training institutions	2
Academia	2
**Total **	**33**

Abbreviations: NGOs, Non-governmental organizations; PWD, persons with disabilities. ^a^ Two interviews were group interviews with participants from the same organization.

 KIs were contacted mainly in-person by a research assistant, as well as by email or by phone. Interviews lasted one hour and were primarily conducted in person at the person’s location of employment or virtually via an institutional Zoom link from December 2022 to April 2023. Two informants with deep knowledge of the historical context participated in a follow-up interview to clarify key insights. Participants provided verbal informed consent to be interviewed, recorded, and to use anonymized excerpts from the interview. Interviews were conducted in English by Ugandan and non-Ugandan researchers. Recordings were transcribed in full and quality checked by the interviewer. Interview transcripts are referenced in the results section with the capital letter I and the corresponding transcript number.

###  Thematic Analysis 

 We used a thematic content analysis methodology to analyze the KIIs.^33^ The sub-components of the prioritization of rehabilitation framework formed initial deductive codes, with inductive codes added iteratively underneath the framework’s sub-components.^33^ The coding was conducted in NVivo12.^34^

 We analyzed the documents identified in the literature review by reading them in full and taking notes to extract key findings. We used the “What’s the problem represented to be?” approach to document analysis,^35^ interrogating how policy and programmatic documents framed the underlying problem definition as it related to rehabilitation, how the authors of the document represent the problem, their positionality, and its effects on present understanding of rehabilitation.

 To triangulate across the KII and document findings, we compared the thematic codes generated from the KIIs with our extracted notes from the document review to corroborating our findings. When the KII and literature data conflicted, we utilized the “What’s the problem represented to be” approach to question those differences and to further examine the positionality of the KI(s) and document author(s).

 After analyzing 70% of the data, we made an initial presentation of our preliminary findings at a rehabilitation planning process meeting for the national government which included both study participants and individuals who did not participate in the study but who work on rehabilitation-related topics at the national level. This improved trustworthiness by engaging participants in interpreting and discussing the results in a one-hour group session (eg, “member checking”).^36^

 Additionally, a running memo was kept to document coding observations and to capture interrelationships in the themes.^37^ This was a key input to the development of the concept map.

####  Development of a Thematic Concept Map 

 A concept map is a visual depiction of themes that emerge from a study.^38,39^ Concept maps are an analytical and visualization tool to reduce qualitative data, visualize connections, and embed themes into the broader context in which they were constructed.^38,39^ Following the thematic analysis, we developed a concept map to describe how the themes were connected. Development of the map was an iterative process that involved mapping the final list of themes and subthemes and interrogating their relationships and interconnectedness.^39^ To guide the concept map development, we further applied the “5 Whys” approach to root cause analysis.^40^ This tool helped us examine the cause-and-effect relationships that participants described across themes and to interrogate how these were driving rehabilitation’s prioritization.

## Results

 The health system’s ability to provide rehabilitation services is perceived as limited, making policy solutions complex. Fragmented governance limits collective action to strengthen implementation. Across the health and social sectors, stakeholders have a different understanding of the core problem to address, leading to different policy solutions. These dynamics limit the emergence of a unified coalition for rehabilitation.

 Present-day challenges are driven by the national legacy of rehabilitation as a social and community-based intervention and the shifting influences of transnational actors on rehabilitation’s problem definition. The legacy of CBR within the MoGLSD has created enabling structures and sensitized government actors on rehabilitation’s importance. At the same time, this legacy institutionalized fragmented governance arrangements and divergent problem definitions across the MoH and MoGLSD, who are primarily engaged in rehabilitation policy and service delivery, and other ministries like MoE&S and Ministry of Local Government, who see themselves as secondary players. Finally, limited knowledge or awareness of rehabilitation services among service users and communities, driven both by stigma and a lack of historical access to these services within the health system, reinforces the perception that there are more pressing priorities for government attention. As a result, rehabilitation is a priority, but an unfunded one (I18, I4, I9, I12, I13).^41^ In the words of a KI:

 “*It is not that it is not planned for but because of constrained resources, rehabilitation is done last” *– District KI (I12).

 These findings are depicted in the Figure. The figure demonstrates the layers of challenges facing rehabilitation’s prioritization and implementation. Below, we present our results along the three categories of the analytical framework: structural factors, governance, and problem definition.

**Figure:**
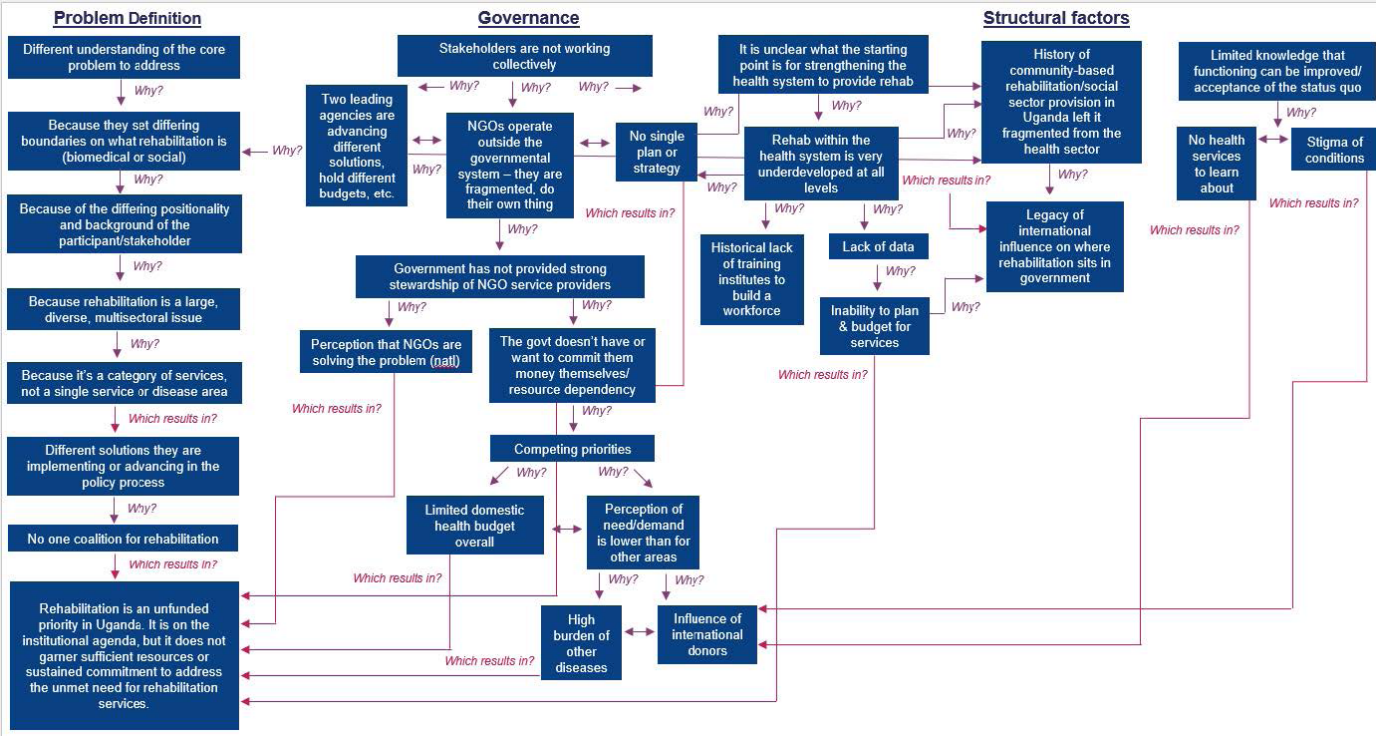
Concept Map for Understanding the Prioritization and Implementation of Rehabilitation in Uganda. Abbreviation: NGOs, Non-governmental organizations.

###  Structural Factors: The Role of the Current Health System 

 Rehabilitation is provided at the secondary and tertiary levels of care. The national government has expanded access to services through orthopedic workshops within regional referral hospitals (I10, I11, I12, I15, I16, I20, I7), by increasing training and degree programs for rehabilitation professionals (I17, I18, I1, I24, I6, I8, I9), and creating rehabilitation positions within the public workforce (I8, I9, I4, I17).^42^ Private hospitals (both for-profit and not-for-profit) have also expanded access to services (I27, I10, I16, I17, I20, I21, I7).

 However, these efforts have focused on hospitals, resulting in geographic and financial accessibility barriers (I26, I27, I29, I10, I15, I16, I19, I1, I21, I25, I23, I2, I3, I5, I9).^42,43^ When services are provided, they often suffer from a lack of trained health workers posted to the hospital (I26, I27, I28, I29, I10, I15, I16, I19, I1, I20, I21, I22, I24, I25, I2, I3, I4, I8, I9) and a limited ability to procure or manufacture assistive technologies (AT) (I13, I16, I17, I19, I20, I21, I24, I3, I7, I8, I9). This results in a perception of intractability of the problem, both for service planners and service users. An non-governmental organization (NGO) worker describes:

 “*If I’m coming from a rural area and the only hospital that I have been referred to is in Kampala (capital city), I need one to look for transport and accommodation and then pay the high fees for that rehabilitation. Many people just end up saying, ‘this is not going to work out, let me just stay with my disability’. […] The services are expensive, they’re not available across the country, infrastructure is there, but equipment is not there, there’s no personnel that are supposed to give these services” *– NGO KI (I27).

 The focus on hospital care is notable because this contrasts to the overall approach of health services in the country, which is to expand access through primary healthcare. An NGO worker describes:

 “*The government has given priorities to certain conditions like malaria and communicable diseases. The government has tried hard to bring these services closer to people at village levels. But when it comes to rehabilitation, you only find these services at regional referral hospitals and selected general hospitals” *– NGO KI (I28).

 Participants therefore interpreted the lack of rehabilitation services at lower levels of the health system as a symptom of relatively lower prioritization compared to curative services. A district official explains:

 “*The service is marginalized at the lower service levels by not even recruiting specialists in those areas. That’s why I’m saying that policy also determines it [lower prioritization]. Though it is a right for everybody”* – District KII (I2).

 Limited investment in rehabilitation services creates path dependency, as the necessary structures to support expanding rehabilitation services remain underdeveloped. For example, investments in treating nodding disease (a childhood epilepsy-like illness associated with physical and mental disability) went un-implemented due to a lack of rehabilitation services.^44^ Similarly, a major weakness of CBR was lack of referral services within the health system.^23^

 Limited rehabilitation service availability has also resulted in limited awareness. In the words of a national government official, “*you cannot be aware of something you have not seen*” (I5). Finally, stigma also reinforces low awareness and limits demand^45–48^; however, most participants suggested that stigmatization of rehabilitation services was improving due to the visibility of PWD in society and the efforts of NGOs and community-based organizations (I27, I28, I29, I11, I15, I18, I1, I22, I1, I20, I22, I4, I25, I5, I23, I24, I25, I3, I4, I8). Self-stigma, or a sense of resignment to one’s fate, was also described by KIs (I2, I7, I27, I26, I1). A national government official explained:

 “*You find an older person whose leg is broken, when you visit him in the hospital, he is like giving up. They say, ‘I am useless, what am I even doing?’. The self-stigma is more entrenched than the community stigma. […] It compromises the push or the assertiveness to demand for the right services”* – National government KI (I7).

 Perceived lack of demand impacts the perception of the severity of the need for rehabilitation, further reducing prioritization. An NGO informant described:

 “*Not many people are asking or requesting for any [rehabilitation] services at the different health facilities closest to them. And now, as Ministry of Health is collecting data and information from these hospitals, they are not getting any data. They’re saying, ‘as a ministry, this area is not our priority, we have not received any cases that would warrant us to invest […] It does definitely affect…how we end up prioritizing these services’” *– NGO KI (I27).

 But lack of data is also evidence of the exclusion of rehabilitation from Uganda’s health management information system and limited population health data estimating the needs of PWD (I27, I28, I29, I11, I13, I14, I15, I16, I18, I23, I24, I25, I7, I8).^20,26,41,42,45,49,50^ This limits the ability to make a compelling case for service expansion:

 “*This is the challenge when it comes to evidence [for] policy decision making. […] And honestly, you find we don’t have the data to support or defend what we bring forward [in the] policy process”* – NGO KI (I29).

 Lack of data on the scope of the problem also limits budget allocation. Informants concurred that there was a lack of government expenditure allocated to rehabilitation (I10, I11, I12, I13, I14, I15, I17, I18, I19, I1, I21, I22, I23, I25, I5, I8), reinforcing all other health systems challenges. A national government official explains:

 “*As we compete for the limited budget, you will end up having some of these areas removed or treated as unfunded partly because of lack of evidence. If I am on the budget committee, why should I allocate money when I don’t know how many people with intellectual disability are there? If I don’t know, you don’t blame me for not giving you money”* – National government KI (I7).

###  Governance: Fragmented Governance Structures and Lack of a Unifying Coalition

 Numerous stakeholders are working to influence rehabilitation (Table 3). Each are advancing problem definitions and solutions from their own perspective, given their distinct interests, backgrounds, and positions.

**Table 3:** Overview of Identified Stakeholders (I28, I29, I10, I19, I21, I26, I29, I11, I13, I15, I19, I1, I6, I7, I4)^42^

**Stakeholder**	**Role**	**Representation for Rehabilitation**	**Summary of Their Power and Position, According to Participants**
**Domestic Stakeholders**			
MoH	Focused on rehabilitation service provision and assistive technology within the health sector. Regulation of rehabilitation healthcare providers.	Public actor: Housed with the Department of Community Health – Disability and Rehabilitation Division.	Strong support within Disability and Rehabilitation Division; however, the MoH overall is perceived to place less emphasis on rehabilitation than curative services with strong donor prioritization/support. Limited representation of rehabilitation in health sector governance documents.^51^
MoGLSD	Focused on improving the lives of and ensuring equality for PWD; enhances community awareness; oversees CBR programmes.	Public actor: Department of Disability and Elderly. Department of Community Development.	Considered a strong advocate for rehabilitation via CBR and community development officers. Interest in revitalizing this approach. Approaches rehabilitation from a psychosocial perspective aligned with PWD rights.
MoE&S	Screening and referral.	Public actor: Special needs department for children with disabilities. Special needs education programme for training teachers.	Focused narrowly on children’s needs and intersection with the education system.
District governments	Include responsible for budget allocation at the district level.	Public actors: Counsellors for the PWD. District Community Development Officers.	Considerable influence on local priority setting and resource allocation.
Parliament	Legal frameworks.	Public actors: Member of Parliament for special needs. Parliamentary health committee.	Considerable influence, jeopardized by a lack of evidence of the problem.
Prime Minister’s Office	Coordination of Ministries.	Public actors: Northern Uganda Rehabilitation – focused on disaster management and refugees.	High influence on financing, and inter-sectoral coordination/fostering policy alignment.
Public and private hospitals	Service provision. Awareness raising through connections to lower-level providers and through community outreach.	Regional referral hospitals Mulago National Referral Hospital. Butabika National Referral Mental Hospital. Private hospitals such as CoRSU, Cheshire Children’s Hospital of Katalemwa, and CURE Children’s Hospital of Uganda.	Rehabilitation service providers are highly supportive. Act as advocates in the policy process by representing the needs of patients and advocating to donors for funding.
DPOs	Umbrella bodies to represent PWD; some organizations are semi-autonomous government agencies while others are civil society organizations.	Various; includes: NUDIPU, National Council for PWD, CBR Alliance, and Uganda Deaf Association.	Representation, but limited perceived power and low capacity. Perceived to be focused on AT and inclusion more than rehabilitation broadly. Limited voice in the design of donor-initiated programs.
Families and patients/PWD	Implementation of home care/self-care.	Carers and rehabilitation service beneficiaries.	Limited skills reliant of healthcare provider coaching/community development officer awareness outreaches.
**Transnational Stakeholders **			
NGOs	Seen as filling gaps in service provision. Many focused in Northern Uganda, previously on civil conflict and now on refugee populations. Often focus on specific conditions or populations.	Rehabilitation Implementing partners.	Limited quality and history of engagement, which gives them variation in influence; some have had sustained service delivery and community engagement programs; others perceived to drop in and out with outdated AT donations.
Donors	Mainly smaller-scale foundations and NGOs. Recent engagement by USAID via the ReLAB-HS consortium.	Rehabilitation development partners.	ReLAB-HS seen as influential in the shift towards integration into the health system.
International organizations	Humanitarian organizations and UNHCR are influential in the integration of rehabilitation into refugee support programming, The WHO is the primary policy influencer and now works closely with MoH.	Rehabilitation focused actors.	WHO is seen as influencing the shift towards health systems integration, via the STARS assessment and Rehab 2030 initiative.

Abbreviations: MoH, Ministry of Health; MoGLSD, Ministry of Gender, Labour, and Social Development; CBR, community-based rehabilitation; PWD, persons with disabilities; MoE&S, Ministry of Education and Sport; DPOs, disabled people’s organization; NUDIPU, National Union of Disabled Persons Uganda; NGOs, Non-governmental organizations; USAID, United States Agency for International Development; WHO, World Health Organization; ReLAB-HS, Learning, Acting, and Building for Rehabilitation in Health Systems; STARS, Systematic Assessment of Rehabilitation Situation; CoRSU, Comprehensive Rehabilitation Services for People with Disability in Uganda; AT, assistive technology; UNHCR, United Nations High Commissioner for Refugees.

 We did not identify a predominant champion for rehabilitation, but instead many different stakeholders all vying for their component of the issue. In the words of a training institute professional:

 “*We need to have a common voice. We need someone who can put us together and share the challenges we are going through and how we can overcome those challenges. We are scattered, everyone is doing his own, we have no one to put us together” – *Training institution KI (I6).

 The result is a fractured coalition. This manifests itself in two ways – fragmentation in donor-funded initiatives and ineffective governance arrangements.

 Specific to donor initiatives, informants agreed that NGOs were playing a key role in service provision, including providing AT and conducting community awareness campaigns (I27, I28, I18, I19, I1, I21, I3, I4, I6, I8, I9). Informants shared that CBR programs and regional referral workshops were functioning largely due to donor support, and that donors played a key role in provision of AT, new technologies, and continuing education programs (I28, I18, I1, I4, I6, I9). Challenges were expressed in matching donor prioritization with local needs, fragmentation, and lack of sustainability. Informants were concerned that donor-funded programs came with their own interests which did not always match the need on ground (I27, I14, I15, I19, I1, I22, I24, I4, I7, I8):

 “*Programs have not been really contextualized. […] but sometimes we are not addressing our own ideas. We really miss out on addressing what are the real needs of our people because the agenda is set elsewhere”* – NGO KI (I19).

 One manifestation is the concentration of donors in post-conflict and refugee populations (I26, I28, I29, I15, I16, I2, I4, I8).^26^ In Northern Uganda, for example, donor-funded NGOs have primarily focused on individuals with war injuries^24,25^; however, a 2020 survey of disability in the Acholi sub-region found that only 6.7% of disability in the region was attributable to war injury.^24^ An NGO worker describes:

 “*Mostly the donors have been supporting [communities in] these conflicts [areas] […] in northern Uganda and west Nile […] and hence, people are able to acquire rehabilitation services, but if they have no conflicts, you find like in central, eastern or western where there are no conflicts, there are very few donors who support rehabilitation”* – NGO KI (I15).

 Further, many Northern Ugandan NGO programs have scaled down or shifted their focus from service delivery to capacity building,^24–26^ with limited sustainability (I27, I16, I19, I22, I5, I8).

 As a result of these narrowly defined interests, donor-funded programs were seen as contributing to fragmentation, both in service delivery and in advocating for a unified vision for rehabilitation’s expansion (I28, I29, I15, I16, I17, I18, I19, I21, I24, I25, I2). Different smaller-scale donors advocating for specific populations could undermine local actors’ ability to make a comprehensive case for support. This contrasts with other health issues in Uganda (supported by large, multilateral donors) which have increasingly moved to basket funding and sector-wide approaches (I21). A hospital staff describes rehabilitation’s fragmented donor funding:

 “*[Donor support] is from a single perspective. That they need to rehabilitate the war victims. […] For example, [NGO] here, has been rehabilitating the war victims. If you lost a limb as a result of a traffic accident, you need rehabilitation, but they will not give you the service because you are not a war victim, and that helps to define rehabilitation in a narrow way”* – Hospital KI (I8).

 In addition, local NGOs and disabled people’s organizations (DPOs) often compete for the same limited donor funding, which can hinder local coalition building and result in competition as organizations with ties to MoH or MoGLSD lobby for funding (I15, I19, I27). An NGO worker explained:

 “*It’s where is most of the money going, which ministry is going to receive money, which one has a bigger budget? And when it gets into that, then it gets political” *– NGO KI (I27).

 Finally, sustainability of donor-funded programs is limited, limiting its influence on the policy process (I27, I14, I12, I11, I15, I16, I17, I18, I19, I21, I24, I3, I25, I4, I5, I6, I7, I9).^25,50^ District and national government officials explained:

 “*They normally run in project framework, the project is just for a short time, so, they don’t have sustainable strategies so that they can push to a level of policy formulation”* – District KI (I12).

 “*They [donors] have an influence, […] some have access to the high level of offices, and they can communicate, but I think they have not been on the system, they have not advocated for the rehabilitation system. They want to offer a service, whether it’s sustainable not sustainable, they just come”* – National government KI (I18).

 Finally, participants suggested that, although the sector is largely dependent on donor funds, that in comparison to infectious diseases and maternal and child health services, rehabilitation was little prioritized by donors (I27, I29, I10, I22, I23, I24, I9).^45^ Several informants also argued that the government’s low prioritization of rehabilitation further exacerbated donor’s limited attention to rehabilitation across sectors (I27, I28, I2, I4, I5):

 “*Normally, when people are coming in, they partner with government, they use the policy […] so once it is not catered for in the policy, then it is a problem, you cannot get funding”* – National government KI (I5).

 Related to the lack of clear government policies for rehabilitation is ineffective governance arrangements, which were seen to fragment a possible coalition of both domestic and transnational actors. The leading ministries for rehabilitation, MoH and MoGLSD, have different mandates, policy frameworks, and visions for how to expand rehabilitation (I26, I10, I29, I16, I17). MoGLSD draws it mandate from constitutional protections for PWD, the PWD Act of 2020, and international treaties for the rights of PWD (I10). In contrast, the MoH draws its mandate from the constitutional right to health, and the National Health Sector Strategic Plan V (2020/2021–2024/2025) (which scantily references rehabilitation in the context of PWDs).^51^ These differing institutional mandates have led to fragmentation, preventing individual efforts from adding up to a strong national consensus and agenda for how to prioritize rehabilitation (I26, I10, I29, I16, I17, I18, I19, I20 I21, I22, I23, I24, I4, I5). A national government representative explained:

 “*There has never been national consensus. We see things happening, we see financers are allocating funds, we see parliament approving whatever, but […] can we have a one national-level decision consensus on rehabilitation? No! We are looking at the same scope, only that we want each of us to know that this is my portion. […] Each ministry has its own goals, I don’t think they align […] though we are serving the same people” *– National government KI (I18).

 However, informants highlighted opportunities to align stakeholders, including the new multisectoral Parish Development Model (I28), a new disability working group across the respective ministries (I5) and increased coordination by the Office of the Prime Minister which is charged with inter-ministerial coordination (I20, I10). One informant pointed to successful advocacy efforts for Uganda’s inclusive education policy – championed by the MoE&S with cross-cutting support built through a multisectoral, institutionalized committee – as a model that could be applied to rehabilitation (I4).

###  Problem Definition: Frame Contestation on How Rehabilitation Is Defined and the Resulting Solutions to Expanding Access to Rehabilitation Services 

 In Uganda, rehabilitation is nearly universally defined as “the improvement of functioning” (I29, I10, I12, I13, I14, I15, I17, I1, I1, I20, I21, I22, I24, I2, I4, I5, I9); however, interrogating that initial definition gives rise to two competing ways of framing the problem. This drives fragmentation, as different government agencies and their stakeholders are advancing related, yet different, solutions to expanding access to rehabilitation services.

 The first framing is a biomedical orientation aligned with rehabilitation as a medical service (I28, I10, I11, I18, I19, I20, I24, I22, I4, I24, I5, I6). Health professional associations, training institutes, hospitals, and the MoH were more frequently aligned to this view, for example:

 “*Health interventions, the biomedical approaches, whereby we have occupational therapy rehabilitation services, physiotherapy rehabilitation services, orthopedic rehabilitation services, largely surgical and assistive technologies, speech language services, optometry, and [..] then hearing” – *National government KI (I18).

 The alternate perspective—represented more frequently by NGOs, MGLSD, PWD organizations, district officials, and academics—is a multisectoral, psychosocial definition, inclusive of social and environmental factors (I26, I27, I12, I14, I15, I16, I17, I19, I21, I22, I23, I7); for example:

 “*You remove the attitudinal barriers, so that you can support a person who has a physical disability, and you want him to access the environment so as you improve the accessibility, […] once the environment is accessible and they can access any service, it is one way of rehabilitating a person” – *NGO KII (I15).

 These two definitions are anchored differentially in the national legacy of rehabilitation and result in different preferences for governance arrangements. Biomedical proponents look to move beyond community-based approaches to increase the integration of rehabilitation into the health system (I4, I25, I24, I20, I26, I27, I10, I11, I14, I19, I21, I24, I3, I8, I9).^41,52^ Solutions to expand rehabilitation include strengthening existing orthopedic workshops (I27, I11, I8), training more rehabilitation health professionals (I28, I18, I22, I4, I14), accessing equipment and AT (I27, I28, I16, I18, I19, I4, I7, I8, I9), improvements in service quality (I28, I19, I7, I26), and increasing financial investment in service delivery (I28, I12, I14, I17, I18, I6, I8). These solutions acknowledge the existing limitations of the health sector but seek to explicitly strengthen its capacity.

 A subset of those espousing the biomedical model provided prevention focused solutions. This view was identified among district and national government decision makers not working directly in rehabilitation (I28, I13, I14, I16, I18, I21). In this framing, the large and growing need for rehabilitation services was also itself a problem requiring preventative solutions:

 “*Since the experts are not there in certain hospitals, you realize that the condition which would have been worked on when the baby is still at may be the pediatric need, this child ends up getting a disability because they did not attend to them” –* NGO KI (I28).

 In contrast, psychosocial proponents seek to build on the legacy of CBR and its community structures to revitalize the community-based model (I26, I28, I10, I15, I18, I19, I20, I22, I24, I4, I7), while improving accessibility for PWD to health services (I17, I19, I23, I6), and emphasizing that PWD should lead solution development (I10, I23, I4, I10). Solutions start from patients and families, as well as community development officers who operate within social services, and with connections to the health system via referrals. This framing often acknowledged the shortcomings of the health system but saw community support as a solution to overcome them, in some cases bypassing the health system entirely:

 “*[Rehabilitation] should be community owned services because if we have adults who are parents, who are caregivers within the community, they learn many skills in provision of rehab services, so they are able to identify, assess, categorize and register these children in the communities. […] getting a physiotherapist is hard” *– NGO KI (I15).

 These differences highlight an underlying challenge – the depth and breadth of how rehabilitation is defined. In the words of a KI, “*rehabilitation is huge*” (I17). For example, bio-medical proponents often focused on visual, hearing, and orthopedic conditions, emphasizing health systems strengthening solutions:

 “*Let this be incorporated into the mainstream health service delivery. […] To start with regional referral hospitals, out of 17, you might find that not more than seven are having a functional rehabilitation unit. How about the rest?” *– Hospital KI (I8).

 Biomedical proponents who equated rehabilitation with preventable causes—such as road traffic accidents, preventable birth injuries, and non-communicable diseases—were proponents of increasing prevention investments (I28, I13, I14, I16, I18, I21):

 “*We should put in preventive healthcare services […] if we put in place mechanisms for early diagnosis for these infections, and effective case management or treatment, we drastically minimize the need for demand for rehabilitative health services” *– National government (I20).

 In contrast, psychosocial proponents often emphasized mental health, congenital conditions, and intellectual disability as disability-related conditions. They were more likely to suggest awareness raising, community engagement, and the overcoming of stigma as the first steps to improving rehabilitation:

 “*We need to sensitize, we need to empower the community when the community arm is empowered, then the rest can be built on that” *– District KI (I12).

 Proponents of both perspectives agreed on the need to strengthen ties between the health system and community structures. However, each participant described the most urgent challenge and solutions from their own perspective. This leads to a lack of clear and united vision of what the core problem definition is and how to overcome it, even at a technical or operational level. This begs the question – where do these competing frames for rehabilitation originate?

###  Structural Factors: Historical Context of Rehabilitation and its Prioritization 

 The current state of rehabilitation services within the health system, its fragmented governance, and competing frames all stem from historical legacies (I7, I4). Tracing the historical evolution of rehabilitation in Uganda elucidates this, beginning with the history of CBR and leading up to present day shifts in how rehabilitation is integrated into the health system.

###  Development of CBR Programming 

 CBR programmes have a long history in Uganda. A national government participant describes the origins:

 “*Initially, we were defining disability in terms of the physical body […] that is way back in the 60’s, later in the 70’s. A lot of debates between WHO and African countries emerged. […] Uganda among other countries said there are other environmental factors, there are other community-based factors beyond the medical model so CBI [Community Based Impairment] emerged in the late 70’s, and it was picked up very strongly in the 80’s, and as a country there was a CBR [Community Based Rehabilitation] strategy” – *National government KI (I7).

 In 1992, the Norwegian Association of the Disabled (NAD)^20^ partnered with the MoGLSD to implement CBR (I4, I7). CBR focused on a community-based, psychosocial approach to providing rehabilitation, initially focused on children with disabilities and expanding under the remit of the MoGLSD (I4, I7). An informant describes the intent of the program:

 “*Their [NAD and MoGLSD ] intention was to train community development officers into identifying disability, supporting the families on early intervention, making referrals to the health services, and welfare services, and doing physical or practical rehabilitation at family level”* – Training institution KI (I4)

 Over time, CBR was seen to strengthen both community and government capacities to provide rehabilitative care:

 “*We had a cadre of rehabilitation officers in all districts across the country […] who knew how to do assessment, who knew how to do referral, who knew how to teach a mother about triggers of disability […] it was a game changer in terms of making mothers “doctors” of their own children and individuals themselves” *– National government KI (I7).

 However, CBR faced implementation and sustainability challenges that led to its collapse. At its height, fragmented partner engagement undermined CBR, with a 2005 evaluation explaining: “it demands a lot of resources for coordination among the various development partner organizations (DPOs) in order to maintain strength and influence in planning and monitoring of the CBR programme outcomes” (p. 3).^23^ An additional sustainability barrier was a lack of referral facilities within the health system, including few physiotherapists.^23,26^

 Eventually, CBR was fully mainstreamed into government systems. An informant recalled:

 “*The project funding ended, and it was now integrated into the national structure. So, that is how it came to a point whereby they created the position of the community development officer, rehabilitation. So that government can recruit those people and be able to continue with the activities as a national program” *– National government KI (I7).

 However, after this transition, and over the next 10-15 years, the CBR program gradually faded as the workforce was not sustained (I4, I7).

###  Global Shifts Towards Integrating Rehabilitation in Health Systems 

 Similar to transnational actor’s historical influence on CBR, the present focus on rehabilitation’s integration into the health system was seen by KIs as being partly driven by the WHO’s current focus of integrating rehabilitation into health systems (I4, I7, I8):

 “*The current move, that is supported by WHO, is defining rehabilitation from the medical perspective. I know that Uganda is trying to harmonize how to take care of both medical and social model of defining rehabilitation” *– Training institute KI (I4).

 This push has been further cemented by the recent influence of the Learning, Acting, and Building for Rehabilitation in Health Systems (ReLAB-HS) consortium and other partners aligned with the WHO’s Rehabilitation 2030 Initiative. For example, a recent Systematic Assessment of Rehabilitation Situation (STARS) process firmly situated rehabilitation within the health sector. A national KI describes progress on this process:

 “*We have done what we call STARS assessment, […] to see what is the burden of disability and rehabilitation needs for those disabilities in the country so that it can be costed and becomes a document that we can sell, that we can use for advocacy to raise resources for rehabilitation services but also to guide the country how we can move rehabilitation agenda forward in the country” *– National government KI (I13).

 This is positioning rehabilitation as more integrated within the health sector. However, there is a tension between this integration and continued proponents of the community-based, psychological approach, as described by a KI:

 “*That whole model of community rehabilitation is really struggling, partly due to structural issues, WHO pushing more for the medical model” *– National government KI (I7).

 This has resulted in a key tension – rehabilitation needs a champion; however, there are multiple potential champions with a differential understanding of the problem and solution, drawing their legitimacy from different legacies of rehabilitation in global policy discourse.

## Discussion

 This study identified that rehabilitation is considered a priority, but an unfunded priority, in Uganda. Expanding access to rehabilitative services is on the institutional agenda of government agencies engaged in the policy-making and delivery of rehabilitation. However, rehabilitation does not garner sufficient financial or human resources or sustained political commitment commensurate with addressing unmet needs.

 Several interrelated dynamics contribute. The national legacy of rehabilitation within social programs instead of the health sector has institutionalized fragmented governance arrangements. Different governmental and non-governmental actors are working within this fragmented system to advance differential problem definitions and solutions for expanding rehabilitation. These efforts are anchored in the differing perspectives and positionalities of specific institutions. They are gradually strengthening service delivery but have failed to produce a unified vision for expanding rehabilitation services which is needed to elevate the issue onto the policy decision agenda. This domestic dynamic is further underscored by the dominance of transnational actors, both in providing rehabilitation services through domestic NGOs and in shaping which components of the health sector receive financing. These dynamics undermine the emergence of a unified coalition and the development of a cohesive policy proposal.

 The analytical framework for the prioritization of rehabilitation^7^ helped to identify the salient factors influencing the prioritization and implementation of rehabilitation in Uganda. Our case study highlights the interactive and reinforcing relationship that the framework’s components exert on shaping rehabilitation’s prioritization.

 This case also highlights the importance of historical context in understanding present-day prioritization and implementation. Tracing the historical evolution of rehabilitation demonstrated the influence of the WHO over time in multiple different frames. It also highlighted fragmentation in policy and service delivery. Informants often provided conflicting information about whether past and current services were still being provided and whether programs were operational, surfacing a lack of interlinkages even within key actors.

 In Uganda, these challenges are not unique to rehabilitation – rather, the challenges limiting rehabilitation’s prioritization and implementation could be viewed as symptoms of the larger health system experiencing the same challenges. This includes limited domestic funding^53^ and challenges working across a decentralized system^27^ considerably dependent on external financial resources. However, perhaps because rehabilitation is “last in line,” it experiences these systemic health systems challenges acutely. Even more challenging, rehabilitation is also subject to common challenges for gaining traction of the prioritization of multi-sectoral problems including a lack of credible indicators, fragmented multisectoral governing coalitions, and unconvincing frames.^10-14^

 Despite these challenges, this case study points to several strategic considerations for the strengthening of prioritization and implementation of rehabilitation services in Uganda.^55^ The first is increasing available rehabilitation data to quantify population needs for rehabilitation services, including population estimates on the unmet need for rehabilitation, effectiveness data on services provided in the Ugandan context, and cost-effectiveness analysis. Evidence from drowning prevention, maternal mortality, and nutrition emphasizes the importance of credible indicators to justify funding allocations.^56-58,61^ In contrast, prioritization of non-communicable diseases, mental health, and violence against children has been weakened by a lack of effective indicators.^11,13,62^

 Second, the development of a rehabilitation strategic plan could unite stakeholders. A key tension is who would act as a policy champion—grounding a strategic plan in a biomedical framing could provide focus, but it risks alienating stakeholders with a psychosocial viewpoint. Evidence from maternal mortality, nutrition, road safety, and drowning preventions emphasizes the importance of champions and policy community cohesion^12,56-58^; in contrast, the prioritization of emergency care has been weakened by a lack of advocates outside the health sector.^59^

 This tension is similarly reflected in worldwide definitions that focus on optimizing functioning of individuals within their environment, emphasizing both health and environmental factors.^60^ For example, a 2022 Delphi study generated a consensus-based definition of rehabilitation similar to the WHO’s^3^ and focused on a multi-modal, person centered process targeting a person’s capacity and contextual factors to optimize functioning.^54^ However, the interventions under this definition are vast and varied, as are the policy solutions for each of these services and populations. A key question is whether rehabilitation advocates can unite around a common framing that is broad enough for a multi-stakeholder coalition but narrow enough to make a specific policy “ask.”

 This case study also demonstrates the importance of framing in the health policy process and how framing (the way a problem is understood and communicated) influences both governance and structural factors. Frames represent a socially constructed view of the world, and divergent framing often underpins policy contestation.^63,64^ Evidence from violence against children, mental health, and non-communicable diseases align with our findings, demonstrating the influence of transnational actors in shaping policy frames, potential solutions, and governance structures.^11,13,62^ In this case, the global re-framing of rehabilitation was an underlying factor in national-level frame contestation, highlighting both the power that global elites have in influencing frames^65^ as well as the agency of domestic actors in adapting and deploying these frames to serve their interests. This frame contestation represents a considerable barrier to prioritization of rehabilitation services in Uganda.

 Finally, this case study points to the challenge of taking rehabilitation services as a unit when engaging in policy advocacy. Different types of rehabilitation services are at different levels of relative maturity or neglect in the system. The health challenges of the 21st century require an integrated, people-centered care approach that places the individual, rather than a collection of individual health needs, at the center.^66^ Building on the people-centered legacy of CBR, aligning it with new multisectoral movements to achieve community-based inclusive development,^67^ and centering the needs and voices of PWD in the policy process will be critical – not only to strengthening rehabilitation’s prioritization but also to ensure that rehabilitation meets the needs of end-users.

###  Strengths and Limitations 

 Strengths of this study include its application of a theoretical framework and its use of member checking to validate emerging themes. Limitations included a limited sample of district KIs and not including service users. Our representation of demand-side factors therefore require corroboration from service users.

## Conclusion

 This case study highlights the challenges of prioritizing rehabilitation services in Uganda and identified strategic opportunities for advancing rehabilitation in a context where there are many competing priorities and limited resources. We identified that rehabilitation is considered a priority in Uganda, but it is an unfunded priority. Challenges to the prioritization and implementation of rehabilitation include frame contestation, fragmented domestic and transnational advocacy coalitions, and the legacy of rehabilitation’s separation from the health sector. Our findings demonstrate the challenges in prioritizing emerging, multi-sectoral health areas and present opportunities for strengthening rehabilitation services, both in Uganda and in similar contexts.

## Acknowledgements

 The authors wish to thank Sarah N. Champagne for contributing to the early concept of this study and the IRB application, Robinah Komuhendo and Ronald Tenywa for the support in data collection, and Renata Kepner for assistance in developing the Figure 1 graphic. This manuscript is related to a published policy brief, which can be found on the ReLAB-HS Resource Library, accessible at https://relabhs.org/publications-and-resources/.

## Ethical issues

 This study was deemed exempted as non-human subject research by the Johns Hopkins University Bloomberg School of Public Health Institutional Review Board (IRB No: 18269). This study was approved by the Makerere University College of Health Sciences School of Public Health Research and Ethics Committee (IRB No: SPH-2022-297). This study was approved by the Uganda National Council for Science and Technology (Ref: HS2400ES).

## Competing interests

 Authors declare that they have no competing interests.
